# KCa3.1 Transgene Induction in Murine Intestinal Epithelium Causes Duodenal Chyme Accumulation and Impairs Duodenal Contractility

**DOI:** 10.3390/ijms20051193

**Published:** 2019-03-08

**Authors:** Marta Sofía Valero, Mariano Ramón-Gimenez, Javier Lozano-Gerona, Pablo Delgado-Wicke, Pilar Calmarza, Aida Oliván-Viguera, Víctor López, Ángel-Luis Garcia-Otín, Salvador Valero, Esther Pueyo, Kirk L. Hamilton, Hiroto Miura, Ralf Köhler

**Affiliations:** 1 Department of Pharmacology and Physiology, Universidad de Zaragoza, 22002 Huesca, Spain; 2 Instituto Agroalimentario de Aragón-IA2 (Universidad de Zaragoza-CITA), 50013 Zaragoza, Spain; ilopez@usj.es; 3 Universidad San Jorge, 50830 Villanueva de Gállego, Spain; m.monxo@gmail.com; 4 Instituto Aragonés de Ciencias de la Salud (IACS), 50009 Zaragoza, Spain; jlozanog.iacs@aragon.es (J.L.-G.); algarcia.iacs@aragon.es (A.-L.G.-O.); 5 Unidad de Investigación Traslacional, Hospital Universitario Miguel Servet, Instituto de Investigación Sanitaria (IIS) Aragón, 50009 Zaragoza, Spain; 6 Department of Biology, Universidad Autónoma de Madrid (UAM), 28049 Madrid, Spain pablo.delgado@uam.es; 7 Clinical Biochemistry Service, Hospital Universitario Miguel Servet, 50009 Zaragoza, Spain; mpcalmarza@salud.aragon.es; 8 BSICoS, Aragón Institute of Engineering Research (IA3), IIS-Aragón, University of Zaragoza, 50009 Zaragoza, Spain; aidaolivanviguera@gmail.com (A.O.-V.); epueyo@unizar.es (E.P.); 9 Biomedical Research Networking Center in Bioengineering, Biomaterials and Nanomedicina (CIBER-BBN), 50018 Zaragoza, Spain; 10 Valero Analítica, S.L. 50011 Zaragoza, Spain; salvador.valero@valeroanalitica.com; 11 Department of Physiology, School of Biomedical Sciences, University of Otago, 9054 Dunedin, New Zealand; kirk.hamilton@otago.ac.nz; 12 Department of Physiology and Cell Biology, University of Nevada School of Medicine, Reno, NV 89557, USA; renomiura@gmail.com; 13 Aragón Agency for Research and Development (ARAID), 50009 Zaragoza, Spain

**Keywords:** intermediate-conductance calcium-activated potassium channel, KCa3.1, epithelium, duodenum, contractility, transgenic mice

## Abstract

The epithelial intermediate-conductance calcium/calmodulin-regulated KCa3.1 channel is considered to be a regulator of intestine function by controlling chloride secretion and water/salt balance. Yet, little is known about the functional importance of KCa3.1 in the intestinal epithelium in vivo. Our objective was to determine the impact of epithelial-specific inducible overexpression of a KCa3.1 transgene (KCa3.1+) and of inducible suppression (KCa3.1−) on intestinal homeostasis and function in mice. KCa3.1 overexpression in the duodenal epithelium of doxycycline (DOX)-treated KCa3.1+ mice was 40-fold above the control levels. Overexpression caused an inflated duodenum and doubling of the chyme content. Histology showed conserved architecture of crypts, villi, and smooth muscle. Unaltered proliferating cell nuclear antigen (PCNA) immune reactivity and reduced amounts of terminal deoxynucleotide transferase mediated X-dUTP nick end labeling (TUNEL)-positive apoptotic cells in villi indicated lower epithelial turnover. Myography showed a reduction in the frequency of spontaneous propulsive muscle contractions with no change in amplitude. The amount of stool in the colon was increased and the frequency of colonic contractions was reduced in KCa3.1+ animals. Senicapoc treatment prevented the phenotype. Suppression of KCa3.1 in DOX-treated KCa3.1− mice caused no overt intestinal phenotype. In conclusion, inducible KCa3.1 overexpression alters intestinal functions by increasing the chyme content and reducing spontaneous contractions and epithelial apoptosis. Induction of epithelial KCa3.1 can play a mechanistic role in the process of adaptation of the intestine.

## 1. Introduction

The intermediate-conductance calcium-activated potassium channel (KCa3.1, encoded by the *KCNN4* gene) is constitutively expressed in the intestinal epithelium [1,2,3]. Concerning its physiological role, it is well established that KCa3.1 activation produces membrane hyperpolarization that is needed for the secretion of chloride anions and concomitantly of water into the lumen [3]. From the pharmacological perspective, blockers of KCa3.1 have been proposed to have anti-diarrheic properties, because the classical, molecule blocker of KCa3.1, clotrimazole, mitigated experimental cholera-toxin-induced diarrhea in mice [4]. There is also early evidence that KCa3.1 may be mechanistically important in inflammatory bowel disease, because a lower epithelial KCa3.1 expression has been found in patients with ulcerative colitis (UC) [5], pointing to a disturbed regulation of chloride secretion/absorption in this disease state. Yet, causality remains elusive. KCa3.1 is also upregulated in activated T cells, where its drives T cell proliferation and synthesis of cytokines during the immune response. Alterations of KCa3.1 have been found in experimental acute and chronic inflammatory processes and autoimmune ulcerative colitis [6,7,8].

However, besides the abovementioned classical physiological functions and pathomechanistic roles in inflammation, there is growing evidence that KCa3.1 promotes excessive cell proliferation, contributing to pathological organ remodeling, such as organ fibrosis of the kidney, transplants, lung, trachea, heart, and liver [9,10,11,12,13,14]. Moreover, KCa3.1 is upregulated in several cancers, such as glioblastoma [15], lung cancer [16], and renal cancer [17], suggesting an oncogenic potential of this channel. Therefore, KCa3.1 can be considered to be a regulator of tissue homeostasis and a driver of pathological cell proliferation.

To elucidate physiological or potential pathological roles of KCa3.1 in the intestinal epithelium, we developed two transgenic mouse models, in which channel expression can be manipulated by a doxycycline (DOX)-sensitive genetic switch, which, when turned on by dietary DOX, causes either epithelial-specific transgene KCa3.1 overexpression (KCa3.1+) or gene deletion (KCa3.1−). We hypothesized that the corresponding genetic manipulations of KCa3.1 alter epithelial homeostasis and proliferation and intestine function.

## 2. Results

### 2.1. Murine Model of KCa3.1-Trangene Induction in the Intestinal Epithelium

**Figure 1:**
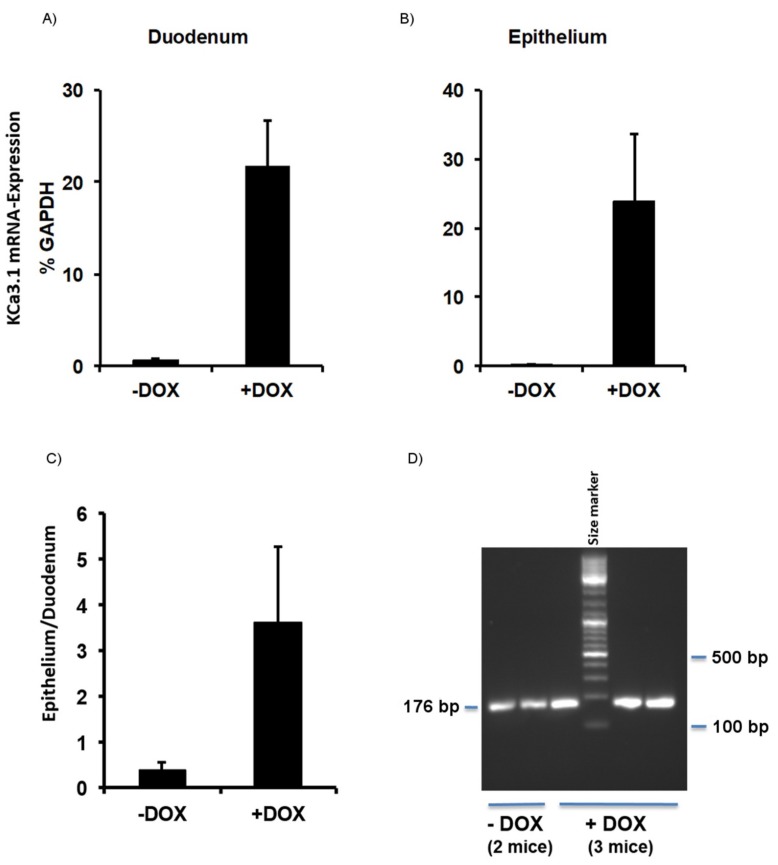
KCa3.1 overexpression in inducible KCa3.1+ mice. Induction of KCa3.1 transgene expression by 2-weeks in doxycycline (DOX)-treated KCa3.1+ mice (+DOX) and untreated KCa3.1+ (−DOX) in (**A**) the duodenum and (**B**) duodenal epithelium measured by qRT-PCR. Expression was normalized to GAPDH expression (% GAPDH). (**C**) The ratio (epithelium/duodenum) of induction of transgene expression. (**D**) Gel electrophoresis showing qRT-PCR. Note the more intense bands in the DOX-treated mice. GAPDH, Glyceraldehyde-3-Phosphate Dehydrogenase; − DOX, untreated KCa3.1+ mice; + DOX, DOX-treated KCa3.1+ mice. The data are means ± SEM, *n* = 2–14 (mice).

The DOX treatment of KCa3.1+ mice (for details, see Section 4) for 2 weeks produced a 22-fold overexpression of KCa3.1 above basal expression levels in the duodenum (Figure 1A). In isolated duodenal epithelium, this overexpression was 40-fold above the levels in the untreated controls (Figure 1B). Epithelial overexpression was fourfold higher than the overexpression found at the duodenal level, demonstrating the strong induction of the KCa3.1-transgene in, particularly, the intestinal epithelium of KCa3.1+ mice (Figure 1C,D).

#### 2.1.1. Phenotype of KCa3.1 Overexpression Mice

DOX-treated KCa3.1+ mice showed a 60% higher water intake during the second week of the DOX treatment when compared with the untreated KCa3.1+ mice (Table 1). The food intake was not significantly different between the groups.

**Figure 2:**
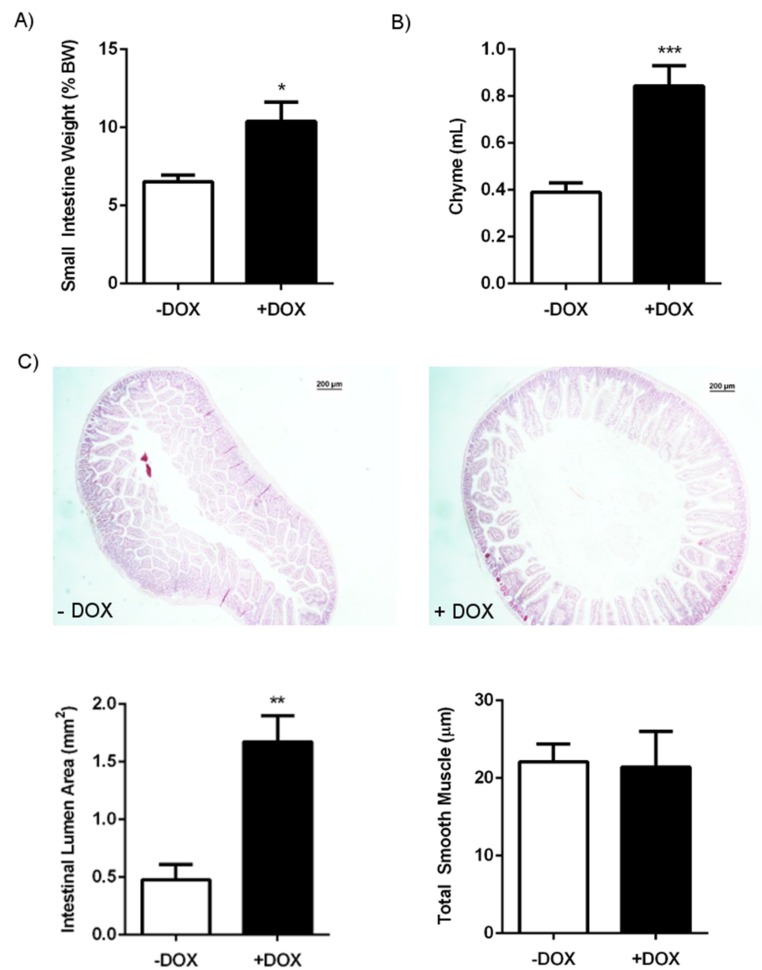
Intestinal content and morphology of the duodenum in DOX-treated (+ DOX) and untreated KCa3.1+ mice (− DOX). (**A**) Small intestine weight. (**B**) Chyme content. (**C**) Hematoxylin–eosin-stained cross-section of the duodenum of the untreated and DOX-treated KCa3.1+ mice, duodenal lumen area and thickness of the longitudinal and circular smooth muscle layers. The data are means ± SEM.; n = 6–15 (mice). * *p* < 0.05, ** *p* < 0.01, *** *p* < 0.001.

Necropsy and histology at sacrifice did not reveal overt alterations of the abdominal organs, with the exception of a visible inflation and higher weight of the small intestine and caecum (Table 2) (Figure 2A). Histology on the DOX-treated and untreated KCa3.1+ mice did not reveal any overt alterations of the epithelium or smooth muscle in the duodenum, such as ulcers, necrosis, rupture of the musclaris, malignancies, villi overgrowth, or loss. Yet, we found a significantly larger duodenal lumen area in the DOX-treated KCa3.1+ mice than in the controls (Figure 2C). There was no difference in the thickness of the muscle layers between the groups (Figure 2C).

**Figure 3:**
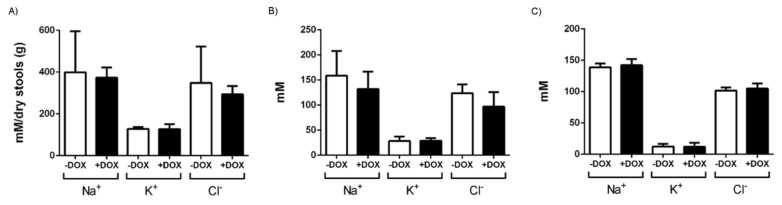
Electrolytic content of stool, chyme, and plasma. Na^+^, K^+^, and Cl^−^ concentrations in (**A**) dried stools, (**B**) chyme, and (**C**) serum of untreated (− DOX) and DOX-treated KCa3.1+ mice (+ DOX). The data are mean ± SEM, *n* = 3–8 (mice).

The total duodenal chyme content at sacrifice was twice as much as in the controls (Figure 2B), thus explaining the difference in diameter and weight. The stool pellets present in the colon of DOX-treated KCa3.1+ mice were of normal shape and had water content similar to those in the controls. Yet, the number of pellets was higher in the DOX-treated KCa3.1+ mice (Table 1). We did not find blood in the chyme and stool samples. The electrolyte composition of chyme, stool, and blood was similar to that in the controls (Figure 3).

#### 2.1.2. Epithelial Homeostasis of the Duodenum

**Figure 4:**
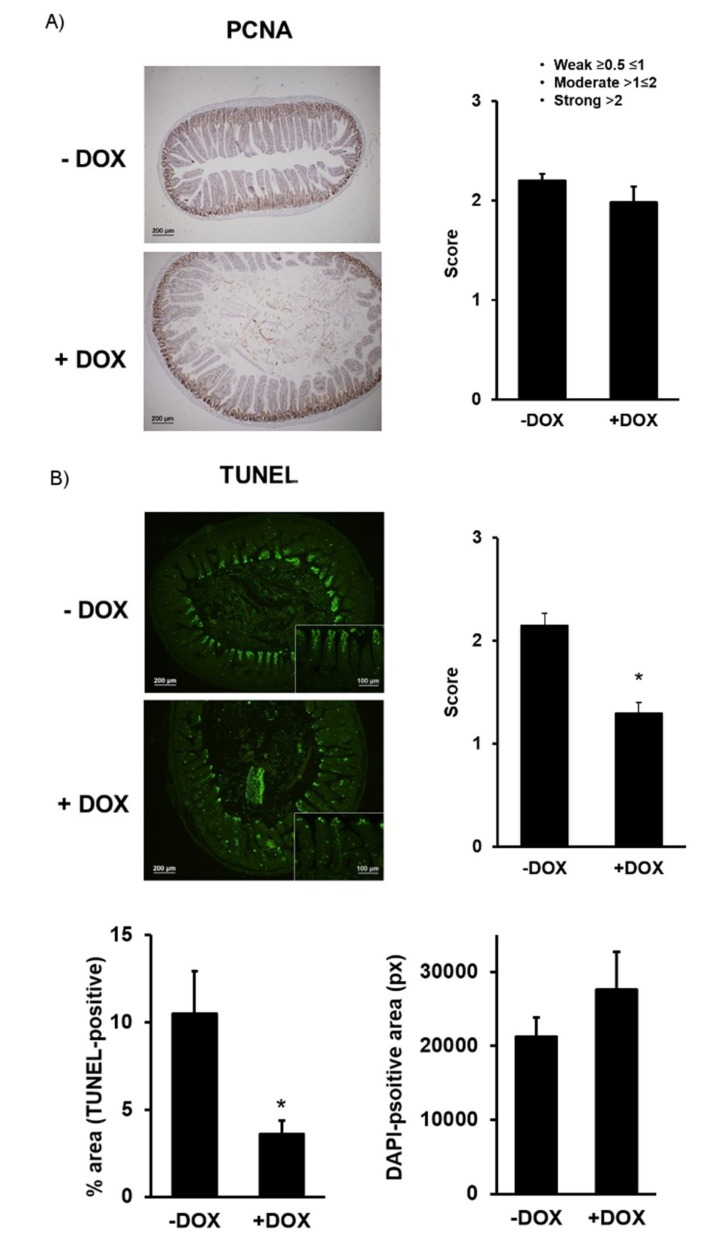
Immune histological examination of proliferating cell nuclear antigen (PCNA) protein expression and terminal deoxynucleotide transferase mediated X-dUTP nick end labeling (TUNEL) in KCa3.1+ mice. (**A**) PCNA-stained and hematoxylin–eosin-counterstained epithelium of untreated (− DOX) and DOX-treated (+ DOX) mice and qualitative scores of intensity. (**B**) Apoptosis in treated and untreated KCa3.1+ mice determined by TUNEL, qualitative scores (panel on right), and quantitative evaluation (lower panels) of signal intensity (% TUNEL-positive; on left) normalized to epithelial area and total 4′,6-Diamidine-2′-phenylindole dihydrochloride (DAPI)-positive area (pixel (px); on right). The data are means ± SEM., *n* = 5 (mice). * *p* < 0.05.

The intestinal epithelium is constantly renewed—a process that involves the proliferation of the basal epithelium forming the villi and a loss of the epithelial cells of the more distal villi segments and a process that involves apoptosis [18]. Our immune histological examination revealed a similar protein expression of the proliferating cell nuclear antigen (PCNA), a DNA-clamp essential for replication and thus an indicator of mitotic activity in the basal epithelial layer (Figure 4A).

Concerning the rates of apoptosis as measured by TUNEL, we found that the distal epithelial cells of villi of the DOX-treated KCa3.1+ showed a significantly lower degree (−40%) of apoptosis (Figure 4B, left panel). The total epithelial area was not different between the groups (Figure 4B, right panel). Together, these results suggest normal duodenal architecture with the exception of a larger lumen area, normal epithelial proliferation rate, and a lower apoptotic activity in the DOX-treated KCa3.1+, suggesting a decreased epithelial turnover.

#### 2.1.3. Alterations of Intestinal Contractility in KCa3.1+ Mice

**Figure 5:**
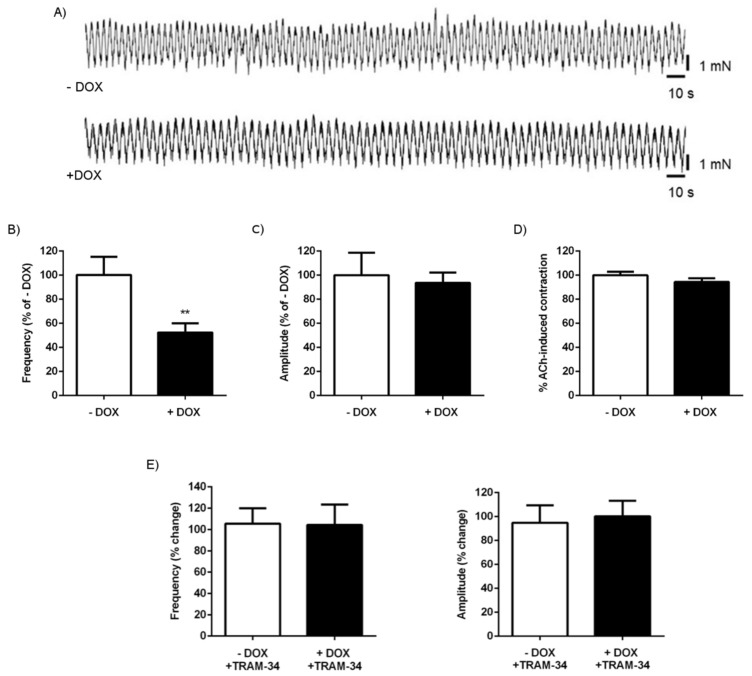
Impact of KCa3.1 transgene induction on duodenal contractility. (**A**) Recording of spontaneous contractions from the longitudinal smooth muscle of untreated (− DOX) and DOX-treated KCa3.1+ mice (+ DOX). (**B**) Frequency and (**C**) amplitude of spontaneous contractions. (**D**) Contractile response to acetylcholine (ACh, at 100 µM). The data are means (% of − DOX) ± SEM. (**E**) There was no impact of TRAM-34 (1 µM) on the frequency and amplitude of the contractions. The data are means (% of – TRAM-34 (before addition TRAM-34) ± SEM.; *n* = 12–16 (mice). ** *p* < 0.01.

The larger duodenal diameter and the larger amount of chyme and stool found in DOX-treated KCa3.1+ mice suggested alterations of intestinal contractility. We, therefore, performed isometric force measurements and determined spontaneous contractions from the longitudinal smooth muscle of the duodenum, as well as acetylcholine (ACh)-induced contractions. As shown in Figure 5A, the spontaneous contractions of the duodenal segments were significantly reduced in the DOX-treated KCa3.1+ mice as compared with the untreated controls. This reduction was due to a lower frequency of contractions (Figure 5B) but not to a reduction of the amplitude of the contractions (Figure 5C).

The magnitude of ACh-induced contractions was similar in the DOX-treated and untreated KCa3.1+ mice (Figure 5D). The KCa3.1 blocker [19] TRAM-34 at 1 µM had no effect on the frequency and amplitude in either group (Figure 5E), suggesting that the transgene function has no direct effect on contractibility.

**Figure 6:**
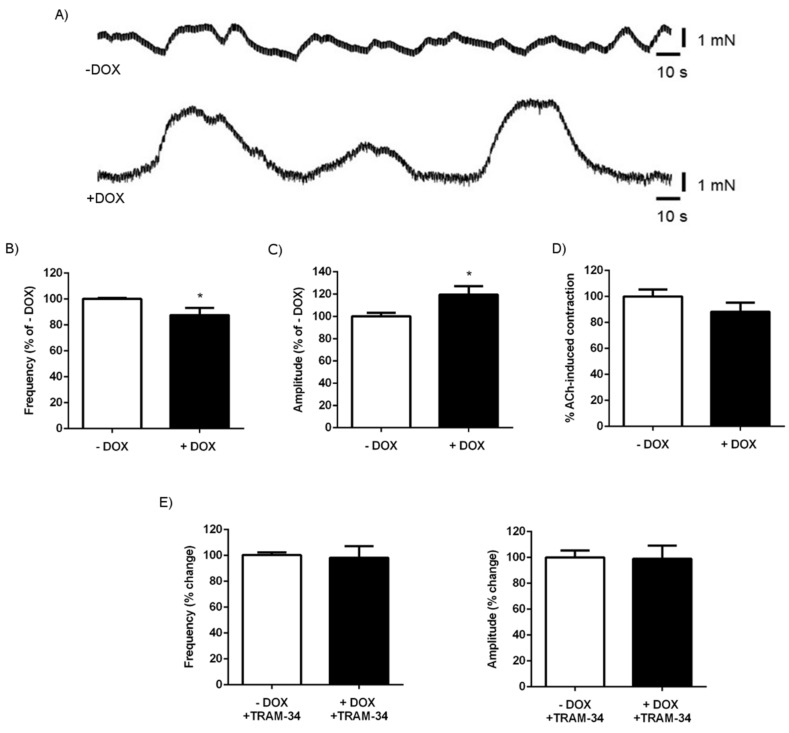
Impact of KCa3.1 induction on colon contractility. (**A**) Recordings of spontaneous contractions in the colonic longitudinal smooth muscle segments of the untreated (− DOX) and DOX-treated KCa3.1+ mice (+ DOX). (**B**) Frequency and (**C**) amplitude of the spontaneous contractions. (**D**) Contractions to acetylcholine (ACh, at 100 µM). The data are given as % of untreated KCa3.1+ mice (− DOX) ± SEM. (**E**) There was no impact of TRAM-34 (1 µM) on the frequency and amplitude of the contractions. The data are means (% of – TRAM-34 (before addition TRAM-34) ± SEM.; *n* = 12–16 (mice). * *p* < 0.05.

We also tested spontaneous and ACh-induced contractions in colon segments (Figure 6). Overall, the contractions were less synchronized than in the duodenum (Figure 6A). Similar to that observed in the duodenal segments, the frequency of the contractions was significantly lower in DOX-treated KCa3.1+ mice than in the untreated controls, although the difference was less pronounced (Figure 6B). Unlike in the duodenum, we found a slightly but significantly larger amplitude of contractions in the colon of the DOX-treated KCa3.1+ mice (Figure 6C). ACh-induced contractions were alike in both groups (Figure 6D). TRAM-34 had no effect on the spontaneous frequency and magnitude of the contractions in either group (Figure 6E).

#### 2.1.4. Senicapoc Treatment

To test whether this intestinal phenotype can be prevented by in vivo KCa3.1 blockade and thus by blocking KCa3.1 transgene function, we treated the mice with DOX and the KCa3.1-blocker, Senicapoc (30 mg/day in chow) because of the proven bioavailability in mice when administered in chow [20]. The treatment prevented changes in water intake and all the morpho-histological and functional alterations in duodenal contractility seen in the DOX-treated KCa3.1+ mice (Table 3), suggesting that the alterations observed in the KCa3.1-overexpressing mice were indeed caused by transgene function.

#### 2.1.5. KCa3.1 Suppression in Intestinal Epithelium

**Figure 7:**
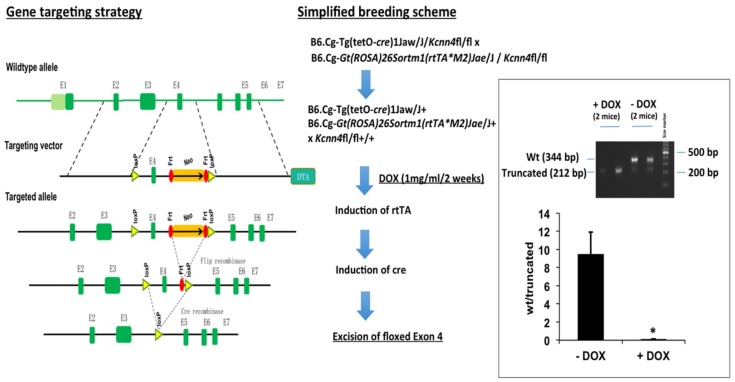
Gene targeting strategy and generation of epithelium-specific inducible KCa3.1− mice.

We also tested the impact of the loss of epithelial KCa3.1 in conditional KCa3.1−. The treatment of conditional KCa3.1− (for details, see Section 4.1) with DOX for 2 weeks caused CRE-mediated excision of the channel pore-encoding exon 4 of the *Kcnn4* gene and the appearance of a shorter mRNA lacking exon 4 as determined by qRT-PCR on the duodenum and isolated epithelium (Figure 7).

**Figure 8:**
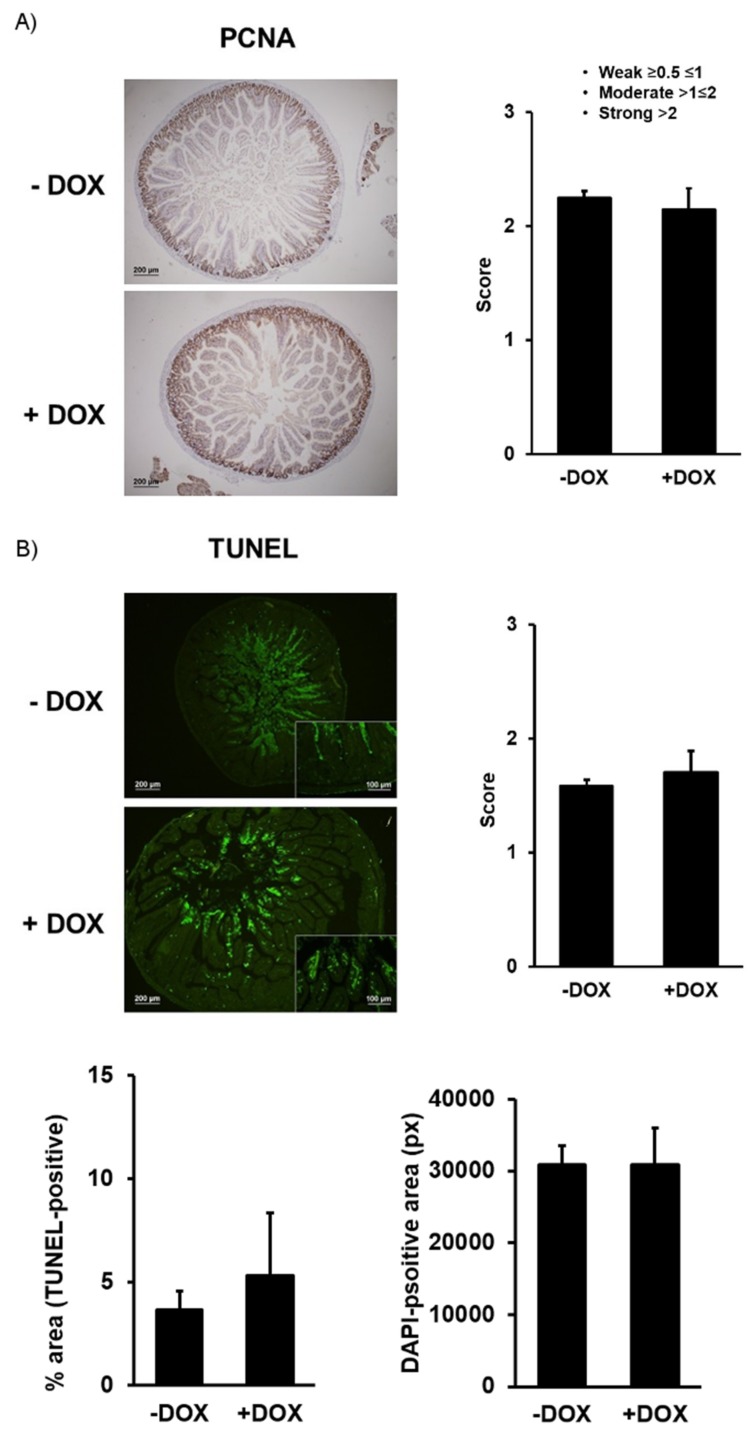
Immune histological examination of PCNA protein expression and TUNEL in KCa3.1− mice. (**A**) PCNA-stained and hematoxylin–eosin-counterstained epithelium of the untreated (− DOX) and DOX-treated KCa3.1− (+ DOX) mice and qualitative scores of intensity. (**B**) Apoptosis in the treated and untreated KCa3.1− mice determined by TUNEL, qualitative scores (panel on right), and quantitative evaluation (lower panels of signal intensity (% TUNEL-positive; on left) normalized to epithelial area and total DAPI-positive area (pixel (px); on right)). The data are means ± SEM., *n* = 4 (mice).

In contrast to the DOX-treated conditional KCa3.1+, loss of the channel did not produce any overt phenotype whatsoever. Moreover, all the morphological and histological parameters, chyme and feces amounts, and duodenal and colonic contractility were alike in the DOX-treated and untreated groups (Table 4). Also, the immune histological examination of PCNA expression (Figure 8A) and the TUNEL assay did not reveal differences in the proliferative activity or rates of apoptosis or size of the epithelial tissue (Figure 8B). These data show that, in contrast to KCa3.1 overexpression, genetically encoded short-term loss of expression of a functional epithelial KCa3.1 in this model has no impact on intestinal function.

## 3. Discussion

Epithelial KCa3.1 channels are thought to play physiological roles in water and chloride transport in the intestine [3] and pathological roles in organ remodeling, inflammation, and cancer as a pro-proliferative and potentially oncogenic channel [15,21,22]. Concerning physiological and pathophysiological roles in the intestinal epithelium in vivo, insight is still limited, perhaps with the exception of an efficacy of KCa3.1 inhibition to treat cholera-toxin-induced diarrhea in mice [4]. We, therefore, developed a novel in vivo approach of genetically encoded and inducible overexpression or suppression of KCa3.1 in the intestinal epithelium. Our main findings are that sub-chronic two-week overexpression of KCa3.1 in the intestinal epithelium caused duodenal chyme accumulation and reduced spontaneous contractions of duodenum. This can be suppressed by pharmacological channel blockade in vivo. Concerning KCa3.1 as a potential pro-proliferative mediator, KCa3.1 overexpression did not change epithelial proliferation but lowered the rates of apoptosis, suggesting decreased turnover, and thereby, an impact of sub-chronic KCa3.1 overexpression on intestinal tissue homeostasis. In contrast to overexpression, sub-chronic suppression of functional KCa3.1 expression had no impact on duodenal and colonic functions. Together, by testing two new genetic models of experimental manipulation of KCa3.1 expression levels, we suggest that sub-chronic KCa3.1-overexpression is capable of altering intestinal homeostasis and transport functions.

Until now, a genetic model of inducible and epithelium-specific KCa3.1 overexpression did not exist. Our genetic KCa3.1+ model consisting of a murine *Kcnn4* transgene under the control of the epithelium-specific DOX-sensitive transactivator (TET-on system) proved to be functional, as we found a solid 22-fold overexpression at the duodenal level and a 40-fold overexpression in the isolated epithelium, fostering tissue specificity of transgene expression in the epithelium (Figure 1B). Overexpression in non-epithelial tissues, such as smooth muscle, skeletal muscle, or nerve and endocrine systems, does not occur in this inducer strain [23].

The first major finding of the present study was that overexpression of epithelium-specific KCa3.1 caused an overt intestinal phenotype with a visibly inflated and heavier duodenum, caused by a substantial accumulation of chyme (Figure 2C). The caecum was also heavier, and the amount of stool pellets present in the colon was increased, suggesting alterations, either of absorption, water/salt imbalance, tissue homeostasis, or intestinal contractility.

In relation to water/salt balance, we did not observe alterations of the electrolyte concentrations in the chyme, stool, or blood, and the water content of the stool was normal (Figure 3), suggesting no overt electrolyte imbalance and dehydration as a result of higher K channel functions, related chloride secretion, and corresponding water movements. However, alterations of chyme electrolytes may have been masked by higher water consumption and the larger chyme content. Still, future cell biological studies focusing on electrolyte transport in vitro may provide deeper insight into the cellular consequences of KCa3.1 overexpression that are beyond the scope of the present in vivo study. 

Concerning KCa3.1 as a channel with tissue homeostasis regulating pro-proliferative function, our histological and immune histological studies excluded tissue hyperplasia, villi overgrowth, or other alterations in the tissue architecture that could explain the visible morpho-anatomical phenotype. Yet, we revealed a decreased rate of apoptotic cells in the distal villi that may represent a trend towards a slower cell turnover and thus points to the role of KCa3.1 in the regulation of intestinal tissue homeostasis.

The second major finding of our study was the reduced frequency of spontaneous duodenal and colonic contractions in KCa3.1-overexpressing mice (Figure 5) that may be the likely cause of chyme and stool accumulation. Yet, when thinking on the underlying cellular mechanisms, this was at first glance surprising, because epithelial KCa3.1 has not been related to control of muscle contractility so far. However, this may still be possible if one speculates that higher K secretion from strongly and perhaps non-polarized KCa3.1-overexpressing epithelium increases the local interstitial K concentrations. One would, thus, expect larger or smaller contractions or contractions with increased frequency, because elevations of extracellular K concentration can have, at first sight, a paradoxical relaxing effect (via stimulation of hyperpolarizing inward-rectifying K channels) or if large, a depolarizing and pro-contractile effect on the duodenal smooth muscle layer. One may also speculate that the higher interstitial K concentration may also affect the activity of enteric neurons and thus the frequency of the contraction. To determine which possibility was more likely, we tested the effects of a KCa3.1-blocker and found that the inhibition of KCa3.1 did not alter the frequency or amplitude of a spontaneous contraction in either KCa3.1-overexpressing mice or untreated mice. This suggests that the lower frequency is not a result of a direct impact of epithelial KCa3.1 transgene function on smooth muscle contractility. Instead, it may reflect a secondary, adaptive phenomenon, perhaps similar to epithelial disturbance, because the lower frequency would slow down propulsion and transit (leading to the observed chyme and stool accumulation) and, thereby, increase absorption time. This possible scenario is further supported by the results of the Senicapoc trial, which demonstrate that channel inhibition in vivo prevents the complete intestinal phenotype, i.e., the lower frequency of spontaneous contractions, the duodenal lumen enlargement, chyme accumulation, and increased stool amounts and water intake. Again, future cell biological studies will help to characterize the nature of the epithelial disturbance causing these alterations. Yet, we can exclude epithelial cell destruction, depletion or rupture, inflammation, and major electrolyte imbalances as primary causes.

Concerning the inducible epithelium-specific deletion model, we indeed found that the model was functional as DOX-induced CRE-mediated excision of the pore-encoding exon 4 occurred at the epithelial levels. However, the DOX-treated mice did not develop any intestinal or other overt phenotype. This was perhaps also to be expected, because life-long loss of KCa3.1 in complete KCa3.1-KO [24] does not show an overt phenotype at the intestinal level or epithelial level but shows endothelial dysfunction [25], defects in erythrocyte volume regulation and adaptive and slowly progressing splenomegaly [24], behavioral alterations [26] and the development of less experimental fibrosis [9], brain damage post experimental mean cerebral artery occlusion [27], and tracheal transplant obstruction [13].

In conclusion, our in vivo study on novel genetic models of inducible KCa3.1 overexpression or suppression identifies epithelial KCa3.1 overexpression as a trigger for chyme accumulation and the reduction of spontaneous contractility. Our findings can benefit the understanding of the cellular mechanisms of small intestine adaptation [28] to surgery and mechanisms to increase nutrient and water absorption in general.

## 4. Materials and Methods 

### 4.1. Epithelium-Specific Inducible KCa3.1+ and Inducible KCa3.1− Mice

Overexpressors: Our TRE-Tg *Kcnn4* mice were generated at Unitech Co., Ltd. (Chiba, Japan). Briefly, we constructed a tetracycline-regulated Kcnn4 expression construct by subcloning PCR-amplified cDNA encoding the open reading frame of *Kcnn4* (gene ID16534) into the pTRE-Tight expression vector (Clontech, Mountain View, CA, USA). The construct was then injected into the pronuclei of fertilized mouse oocytes of the C57BL/6J strain. The putative TRE-Tg*Kcnn4* founders obtained were genotyped by PCR with primers specific for the murine *kcnn4* sequence (*Kcnn4*: F-CAAGCACACTCGAAGGAAGGACTC; R-GGAGATGTCCACCATGGAATTCAC). Then, 2 founders were crossed with wild-type C57BL/6J mice to generate the F1 generation. Furthermore 1 line was inbred over 2–3 generations and subsequently crossed with B6. Cg-Gt(ROSA)26Sortm1(rtTA*M2)Jae/J+. Routine genotyping was performed by using DNA from tail tips, PCR primers (for *Kcnn4*: see above; for ROSA as provided by the supplier (Jackson Laboratory, Bar Harbor, ME USA), and SuperHotTaq Master mix (BIORON GMBH, Ludwigshafen, Germany). The cycle program was as follows: 94 °C for 2 min, followed by 35 cycles at 94 °C for 20 s, 56 °C for 30 s, 72 °C for 30 s and cooling to 10 °C. PCR products for wt (330 bp) and *kcnn4* (139 bp) were analyzed by gel electrophoresis (1.5% agarose, not shown).

For transgene induction, double hemizygous male and female offspring (8–20 weeks old) received doxycycline (DOX, 1 mg/mL water) for 2 weeks. Duodenum segments were fixated in neutral-buffered formaldehyde (4%) or stored at −80 °C for later quantitative PCR analysis. A group of mice received DOX together with Senicapoc-containing chow, giving a dose of 30 mg/kg/day, which is known to produce pharmacologically relevant plasma levels above 100 nM at sacrifice. The expression levels were determined by quantitative RT-PCR. Total RNA was isolated with TriReagent (MCR, Cincinnati, OH, USA) and purified using a RNA Clean-up and Concentration-Micro-Elute kit (Norgen Biotek, Thorold, ON, Canada). Digestion of genomic DNA was done by using the Ambion DNA-free kit (Invitrogen, Carlsbad, CA, USA). The quality and quantity of extracted RNA were determined using the NanoDrop1000 spectrometer (Thermofisher, Waltham, MA, USA). A total of 600 ng of total RNA were reverse-transcribed with Super Script III reverse transcriptase (Invitrogen, Carlsbad, CA, USA) and random hexamers. Then, 10 ng cDNA was amplified using SYBR Select Master Mix and a StepOnePlus Real-Time PCR system (Applied Biosystems, Foster City, CA, USA) and the following primers for *Kcnn4*: F-GTCTGCTGCACAGCTCTCCT; R-TCCTTCCTTCGAGTGTGCTT; expected product size 176 bp). The cycle protocol was as follows: 95 °C for 15 s and 60 °C for 60 s repeated for 40 cycles. The expression levels were normalized to the expression of *Gapdh* as the reference gene (primer: F-AGGGAGATGCTCAGTGTTGG; R-CAATGAATACGGCTACAGCAAC) using the following formula: % *of* *Gapdh* = *Efficiency*(*Gapdh*)^*Cq*(^*^Gapdh^*^)^/*Efficiency*(*Kcnn4*)^*Cq*(*Kcnn4*)^ × 100.

Conditional knockout: *Kcnn4*-floxed mice were generated by Biocytogen, LCC (Worcester, MA USA). Briefly, the *Kcnn4* gene was modified by the insertion of two synthetic loxP sites placed in such a way as to leave the *Kcnn4* gene fully functional (Figure 7). To achieve this, loxP and FRT sites were introduced into the targeting vector: two FRT sites flanking the neo selection cassette were placed 239 bp downstream of exon 4. The SspI-tagged loxP site was inserted into the third intron and the HindIII-tagged loxP site downstream of the second FRT site. The 7.4 kb 5′ homologous arm and the 4.3 kb 3′ homologous arm were introduced into the targeting vector by bacterial artificial chromosomes (BAC) recombination. The Asc I linearized targeting vector was electroporated into C57BL/6J ES cells. Homologous recombinant clones were identified by the hybridization of Ssp I-digested genomic DNA with probe 1 and HindIII0digested genomic DNA with probe 2. The wild-type SspI fragment was 11.2 kb, and the mutant fragment was expected to be 9.5 kb. The wild-type HindIII fragment was 6.6 kb, and the mutant fragment was expected to be 5.5 kb. The mice were produced by standard blastocyst injection procedure. The mice were genotyped by standard PCR on tail tips using the primers *Kcnn4*-A1 LoxP-F TCATCCTCCATAATGCTGCGATCT and *Kcnn4*-A2 LoxP-R CAAGTTTAGGTCTGGTCAAGCCTG, giving products for the wt allele (247 bp (wt) and *Kcnn4*-flox allele (311 bp). Homogenous *Kcnn4*-floxed mice were then crossed with conditional cre-recombinase expressing B6. Cg-Tg(tetO-cre)1Jaw/J (Jackson Laboratory) or Cg-Gt(ROSA)26Sortm1(rtTA*M2)Jae/J+ (Jackson Laboratory). Cre and ROSA strains were genotyped according to the supplier’s protocol. Offspring homogenous for both gene modifications were inbred until mice homogenous for all three modifications (in the following referred to as conditional KCa3.1−) were obtained. 

For epithelial specific suppression, KCa3.1− mice received DOX (1 mg/mL) for 2 weeks. To demonstrate successful deletion of exon 4, mRNA was extracted and reverse-transcribed using the protocols described above. Successful deletion of exon 4 in the duodenum was verified by RT-PCR using the forward primers GTGGCCAAGCTGTACATGA (exon 3) and the reverse primer TCCTTCCTTCGAGTGTGCTT (exon 6). The reaction amplified a truncated fragment (280 bp; lacking exon 4 (136 bp) in the DOX-treated conditional KCa3.1− and the larger wt fragment (416 bp, un-cut by CRE) indicating expression of the wt KCa3.1 or un-cut conditional KCa3.1− in other duodenal cells than the epithelium (Figure 7). The intensity of the small and large fragments per lane was analyzed on black/white-inverted images of electrophoresis gel (1.5% agarose supplemented with Gelred^®^), and the pixel number per fragment was quantified using the histogram function of the ImageJ program. The ratio of the pixel number (large fragment/small fragment) was calculated and represented in the graph of Figure 7.

All the procedures were approved by the Universidad de Zaragoza Animal Ethics Committee (PI27/13; PI28/12; PI37/13/16; PI32/15 (09 October 2015) and in accordance with the ARRIVE guidelines.

### 4.2. Tissue Preparation

After the mice were sacrificed, the gastrointestinal package was removed and placed in ice-cold phosphate-buffered saline (PBS) solution. All the organs (stomach, small intestine, caecum, and large intestine) were weighed. The content of the small intestine was measured and kept for electrolytic studies. The duodenum and colon were cut in segments for different studies. The initial portion was prepared for histopathology. The middle portion was used for myography studies, and the more distal portion was used for qPCR-studies.

### 4.3. Histology

Segments of proximal duodenum were fixed in 4% neutral-buffered formaldehyde, embedded in paraffin blocks and cut into 4-μm-thick sections. They were stained with hematoxylin–eosin and examined. The thickness of the longitudinal and circular smooth muscle layers and the cross-sectional area of the duodenal wall were determined. The thickness was measured at 3 different points using a Nikon Eclipse Ci microscope, a Nikon DS-Ri 1 digital still camera, and the Nikon NIS calibrated digital image analysis system (Nikon, Badhoevedorp, The Netherlands). The cross-sectional area was determined using ImageJ (downloaded from https://imagej.nih.gov/ij/index.html).

Immunohistochemistry of proliferating cell nuclear antigen (PCNA): 5-µm-thick tissue sections were placed on silanized slides. After de-paraffinization, endogenous peroxidase was quenched by immersing the samples in methanol containing 0.03% hydrogen peroxide. Heat antigen retrieval was performed in pH6.5 10 μM citrate-based buffered solution (Dako, Santa Clara, CA, US). In order to prevent non-specific binding, the samples were incubated with Protein Block (Dako). The samples were incubated overnight at 4 °C with mouse monoclonal antibody against PCNA (Cell Signaling, Leiden, The Netherlands, 1:500), followed by incubation with an anti-mouse polymer-based Ig coupled to peroxidase (Cell Signaling) for 30 min at RT. Then, the sections were incubated with 3,3′-diaminobenzidine (DAB) and counterstained with hematoxylin.

Apoptosis was determined by using TUNEL (terminal deoxynucleotide transferase mediated X-dUTP nick end labeling) assay. Briefly, tissue sections (5 µm) were placed on silanized slides. The sections were de-paraffinized and rehydrated and then treated with Proteinase K (20 µg mL^−1^, 15 min, room temperature) following by washing 2 times with PBS. Afterwards, the sections were incubated for 1 h at 37 °C with the in situ Cell Death Detection Kit (Roche Diagnostics GmbH, Roche Applied Science, Mannheim, Germany), according to the manufacturer’s instructions. For nuclear counterstaining, the samples were incubated with Höechst-33258 (Sigma, Darmstadt, Germany) at a final concentration of 1µg/mL and mounted with Vectashield mounting medium (Vector, Burlingame, CA, USA).

The sections were examined with an Olympus BX-61 epifluorescence microscope equipped with filter sets for fluorescence microscopy: ultraviolet (UV, 365 nm, exciting filter UG-1 (ultraviolet glas-1) and blue (450–490 nm, exciting filter BP 490). Photographs were taken with an Olympus CCD DP70 digital camera and processed with the Adobe PhotoShop 7.0 software (Adobe Systems, San Jose, CA, USA). For quantifying and comparing PCNA expression and apoptosis, the duodenal sections were scored by three investigators (R.K., A.-L.G.-O., and A.O.-V.), independently and in a blinded fashion, using an in-house scoring system. The individual scores were averaged. For the quantification of signal intensity, we measured DAPI-positive and TUNEL-positive areas using the Fiji software for image analysis and calculated the percentage of TUNEL-positive areas ((pixels(TUNELpositive)/pixels(DAPI-positive)×100)).

### 4.4. Intestinal Contractility Studies

The middle of the duodenum and colon were immediately washed with cold Ringer Krebs solutions (NaCl 120 mM, KCl 4.7 mM, CaCl_2_ 2.4 mM, MgSO_4_ 1.2 mM, NaHCO_3_ 24.5 mM, KH_2_PO_4_ 1 mM, and glucose 5.6 mM, pH 7.4), cleaned from mesenteric attachment, and cut into segments. Whole segments (10 mm long) were suspended in the direction of longitudinal smooth muscle fibers in an organ bath containing 5 mL of Ringer Krebs, maintained at 37 °C and were continuously gassed with carbogen (95% O_2_ and 5% CO_2_). Muscle tension of each segment were measured isometrically with an initial tension of 9.8 mN, and changes in mechanical activity were amplified, recorded on a computer for analysis (AD Instruments Inc, Milford, MA, USA), and digitized at a sample per second per channel.

After an adaptation period (60 min), we recorded spontaneous contractions and tested the effect of TRAM-34 (1 µM, 15 min exposure time), a KCa3.1 blocker, and of acetylcholine (ACh, 100 µM, exposure time 3 min), a cholinergic agonist, on intestinal contractility. To analyze spontaneous contractions, we measured the mean frequency (contractions per minute) and amplitude of force (in gram) over 5 min. The ACh-induced motor responses were measured as integrated mechanical activity per second during the first 3 min of ACh response, minus the integrated area per second of spontaneous contractions. The data are given as g/s and normalized to weight (g) of wet tissue. The responses were normalized to responses in the control mice (% of − DOX). The amplitude and frequency of the spontaneous contractions in the presence of TRAM-34 were expressed as a percentage of the control (− TRAM-34, before addition of TRAM-34). 

### 4.5. Hydroelectrolytic Determination in Serum, Chyme, and Stools

Stools pellets found in the colon were weighed (Wet Stools), dried for 48 h at room temperature, and then weighed again (Dry Stools). Thereafter, they were re-suspended on 1 mL of deionized water and heated to 65 °C for 60 min. The suspensions were centrifuged at 12,000 g for 5 min. The supernatant was filtered through Millex 0.22 µm filter units, and Na^+^, K^+^, and Cl^−^ assays were performed using a standard electrolyte analyzer equipped with ion selective electrodes. The calculation of water content was performed using the following formula: % water = (wet stool (mg) − dry stool (mg)) ×100/wet stool (mg). The blood and chyme samples were also centrifuged at 3500 g for 10 min at 4 °C to separate the serum or supernatant, and the concentration of Na^+^, K^+^ and Cl^−^ were determined.

### 4.6. Statistical Analyses

The data are expressed as mean ± SEM. Normal distribution of the sample was assessed by Shapiro–Wilk test. Student’s *T* test or Mann–Whitney test were performed to compare between 2 groups (− DOX vs + DOX, in the different conditions). *p*-values of <0.05 were considered statistically significant. The statistical analysis of the data was performed using GraphPad Prism version 6.0 software (San Diego, CA, US). 

## Figures and Tables

**Table 1:** Changes in water and food consumption and amounts and water content of stools. − DOX, untreated KCa3.1+ mice. + DOX, doxycycline-treated KCa3.1+ mice.

	−DOX		+DOX		*p* vs. − DOX
	Mean + SEM	*n* (mice)	Mean + SEM	*n* (mice)	
Water intake 1st week (mL/day)	4.7 ± 0.3	33	4.1 ± 0.4	34	ns
Water intake 2nd week (mL/day)	4.2 ± 0.3	33	6.8 ± 0.6	34	0.0005
Food intake (g)	1.4 ± 0.2	11	1.7 ± 0.2	9	ns
**Stool pellets:**					
Wet weight (mg)	97 ± 10	11	140 ± 11	14	0.009
Dry weight (mg)	27 ± 3	11	40 ± 3	14	0.009
Water content (%)	72 ± 1	11	71 ± 1	14	ns

ns: not significant.

**Table 2:** Effects of overexpression of KCa3.1 on the weight of the gastrointestinal package, small intestine, stomach, caecum, and colon. Organ weights were normalized to body weight and expressed as a percentage of body weight ((g organ/g BW)×100). − DOX, untreated KCa3.1+ mice. + DOX, doxycycline-treated KCa3.1+ mice.

	− DOX		+ DOX		*p* vs. − DOX
	Mean ± SEM (g organ/g BW) × 100	*n* (mice)	Mean ± SEM	*n* (mice)	
**Organography**					
GI package	12.7 ± 0.6	12	17.5 ± 1.8	8	0.0328
Small intestine	6.5 ± 0.4	12	10.4 ± 1.2	8	0.0120
Stomach	1.7 ± 0.2	10	2.1 ± 0.3	8	ns
Caecum	1.6 ± 0.1	12	4.9 ± 0.6	8	<0.0001
Colon	1.6 ± 0.2	12	1.5 ± 0.1	8	ns

GI: gastrointestinal, BW: body weight.

**Table 3:** In vivo Senicapoc treatment prevents the intestinal phenotype in DOX-treated KCa3.1+ mice. The data are expressed as % of untreated KCa3.1+ mice (− DOX) ± SEM.

	+ DOX + Senicapoc	*n* (mice)	*p* vs. − DOX
	(% of –DOX)		
Water intake 1st week	79 ± 27	4	ns
Water intake 2nd week	98 ± 17	5	ns
Food intake	147 ± 22	5	ns
**Organography**			
GI package	121 ± 9	6	ns
Small intestine	123 ± 17	6	ns
Stomach	120 ± 17	6	ns
Caecum	125 ± 12	6	ns
Colon	100 ± 20	6	ns
Chyme	74 ± 9	6	ns
ILA	105 ± 30	6	ns
**Duodenum contractility**			
Frequency	102 ± 23	6	ns
Amplitude	90 ± 13	6	ns
**Colon contractility**			
Frequency	96 ± 4	6	ns
Amplitude	105 ± 3	6	ns

GI: gastrointestinal, ILA: small intestine lumen area, ns: not significant.

**Table 4:** Suppression of KCa3.1 in KCa3.1− mice causes no overt intestinal phenotype. Organ weights were normalized to body weight (BW) and expressed as a percentage of BW ((g organ/g BW) × 100). The intestinal contractility data are given as % of control (untreated KCa3.1− mice (− DOX)).

	− DOX		+ DOX		*p* vs. − DOX
	Mean ± SEM	*n* (mice)	Mean ± SEM	*n* (mice)	
Water intake 1st week (mL/d) Water intake 2nd week (mL/d) Food intake (g/d) **Stool pellets:**	1.8 ± 0.1 0.9 ± 0.1 1.4 ± 0.2	9 9 9	1.9 ± 1.0 0.9 ± 0.1 1.7 ± 0.2	12 12 12	ns ns ns
Wet weight (mg)	236 ± 31	9	213 ± 29	15	ns
Dry weight (mg)	40.7 ± 6.3	9	36.0 ± 5.2	15	ns
Water content (%)	81.3 ± 0.5	9	81.5 ± 1.3	15	ns
**Organography**					
GI package	19.8 ± 1.0	9	21.8 ± 0.3	15	ns
Small intestine	6.3 ± 0.3	9	7.2 ± 0.4	15	ns
Stomach	1.8 ± 0.1	9	2.1 ± 0.1	15	ns
Caecum	2.6 ± 0.1	9	3.1 ± 0.2	15	ns
Colon	1.9 ± 0.1	9	2.0 ± 0.1	15	ns
Chyme (mL)	0.3 ± 0.0	9	0.4 ± 0.0	15	ns
ILA (mm^2^)	0.1 ± 0.0	9	0.2 ± 0.0	15	ns
**Duodenum contractility**					
Frequency (% of − DOX)	100 ± 2	8	99 ± 2	8	ns
Amplitude (% of − DOX)	100 ± 16	8	99 ± 13	8	ns
Frequency (% change-TRAM-34)	103 ± 1	8	100 ± 3	8	ns
Amplitude (% change-TRAM-34)	99 ± 18	8	98 ± 24	8	ns
**Colon contractility**					
Frequency (% of − DOX)	100 ± 6	8	116 ± 7	8	ns
Amplitude (% of − DOX)	100 ± 17	8	96 ± 19	8	ns
Frequency (% change-TRAM-34)	99 ± 4	8	98 ± 4	8	ns
Amplitude (% change-TRAM-34)	105 ± 20	8	96 ± 12	8	ns

d: day. GI, gastrointestinal. ILA: Small intestine lumen area. ns: not significant.

## Abbreviations

DAPI	4′,6-Diamidine-2′-phenylindole dihydrochloride
DOX	Doxycycline
KCa3.1	Intermediate-conductance calcium-activated potassium channel Type 4
KCa3.1+	Conditional KCa3.1-overexpressing mice
KCa3.1−	Conditional KCa3.1-suppressing mice
*Kcnn4*	Intermediate-conductance calcium-activated potassium channel Type 4 gene
PCNA	proliferating cell nuclear antigen
TUNEL	terminal deoxynucleotide transferase mediated X-dUTP nick end labeling
